# Depressive symptoms are associated with the incidence of frailty in older adults with cardiometabolic diseases: Interaction between depression and diabetes

**DOI:** 10.1371/journal.pone.0360802

**Published:** 2026-10-26

**Authors:** Fumino Kobayashi, Yoshiaki Tamura, Yuji Murao, Kenji Toyoshima, Kazuhito Oba, Remi Kodera, Ai Iizuka, Yuko Chiba, Ayumi Toba, Takumi Hirata, Yumi Shindo, Joji Ishikawa, Atsushi Araki

**Affiliations:** 1 Center for Comprehensive Care and Research for Prefrailty, Tokyo Metropolitan Institute for Geriatrics and Gerontology, Tokyo, Japan; 2 Department of Diabetes, Metabolism and Endocrinology, Tokyo Metropolitan Institute for Geriatrics and Gerontology, Tokyo, Japan; 3 Department of Cardiology, Tokyo Metropolitan Institute for Geriatrics and Gerontology, Tokyo, Japan; 4 Human Care Research Team, Tokyo Metropolitan Institute for Geriatrics and Gerontology, Tokyo, Japan; 5 Center for Cultivating and Enlightenment for Healthy Longevity, Tokyo Metropolitan Institute for Geriatrics and Gerontology, Tokyo, Japan; International University of Health and Welfare, School of Medicine, JAPAN

## Abstract

**Background:**

Older adults with diabetes are prone to frailty and increased mortality.

**Aim:**

In this longitudinal study, we examined the association between depressive symptoms and the incidence of frailty in older people with cardiometabolic diseases. We also investigated whether comorbidities modified the association between depressive symptoms and incident frailty in this patient population.

**Methods:**

We analyzed longitudinal data from 431 outpatients aged 65 years or older with cardiometabolic diseases who visited a frailty clinic from 2015 to 2023. Depressive symptoms were assessed using the Geriatric Depression Scale (GDS)-15. The outcome during follow-up (median, 2.9 years) was incident frailty, as defined by the modified Cardiovascular Health Study (mCHS) criteria or the Kihon Checklist (KCL). Hazard ratios (HRs) and 95% confidence intervals (CIs) for incident frailty were estimated using multivariable Cox regression models.

**Results:**

Depressive symptoms (GDS-15 ≥ 5) were independently associated with incident mCHS- and KCL-defined frailty across all models. The fully adjusted HRs for incident mCHS- and KCL-defined frailty were 1.92 and 2.05, respectively. Stratified analyses demonstrated consistent associations between depressive symptoms and incident frailty in participants with diabetes and hyperglycemia. Significant interactions between depressive symptoms and diabetes were observed for both mCHS- and KCL-defined frailty, whereas no significant interactions were observed for cognitive impairment or physical inactivity.

**Conclusion:**

Depressive symptoms were independently associated with incident frailty, as defined by both the mCHS and KCL criteria, in older outpatients attending a frailty clinic for cardiometabolic diseases. A significant interaction between depressive symptoms and diabetes was observed, suggesting that patients with coexisting depressive symptoms and diabetes may represent a high-risk group for incident frailty. Further studies are needed to determine whether interventions targeting depressive symptoms and diabetes management can reduce the risk of incident frailty.

## Introduction

Managing geriatric syndromes, including frailty, in older adults with cardiometabolic diseases such as diabetes and hypertension is of great importance in clinical practice [1,2]. Older adults with diabetes are prone to frailty [3,4], leading to increased mortality [5,6]. Therefore, early screening and prevention of frailty are crucial for older adults with cardiometabolic diseases.

Older adults with depressive symptoms may become frailty [7], due to social isolation [8], confinement, and low levels of physical activity [9], creating a vicious cycle of frailty [10]. Some longitudinal studies involving community-dwelling older adults have shown that depressive symptoms are risk factors for incident frailty [11]. Conversely, frailty has also been reported to increase the risk of subsequent depression, suggesting a bidirectional relationship between depression and frailty [12]. However, the longitudinal association between baseline depressive symptoms and incident frailty remains unknown in older outpatients with cardiometabolic diseases who are at high risk of frailty.

We previously reported a cross-sectional association between depressive symptoms and prevalent frailty in older outpatients with cardiometabolic diseases, including diabetes and hypertension [13]. Because that study was based on cross-sectional baseline data, it could not determine the temporal relationship between depressive symptoms and frailty. The present longitudinal study extends our previous work by using the follow-up data to specifically focus on one direction of this relationship by examining whether baseline depressive symptoms predict the subsequent incidence of frailty in older adults with cardiometabolic diseases. In addition, it remains unclear whether comorbidities commonly associated with depressive symptoms modify the association between baseline depressive symptoms and incident frailty. Although depressive symptoms in older adults have been reported to be associated with diabetes [14], cardiovascular disease [15], cognitive impairment [16], polypharmacy [17], and reduced social support [8], few longitudinal studies have examined whether the coexistence of depressive symptoms and these comorbidities is associated with an increased risk of incident frailty. Specifically, depression in patients with diabetes may lead to frailty through hyperglycemia and diabetic complications [18,19]. We hypothesized that the coexistence of depressive symptoms and diabetes would synergistically increase the risk of incident frailty because these conditions may share common mechanisms contributing to frailty, including inflammation, insulin resistance, and activation of the hypothalamic–pituitary–adrenal axis in response to stress [18]. Interactions with other comorbidities and clinical or social factors, including hypertension, dyslipidemia, cognitive impairment, polypharmacy, physical activity, and social networks, were evaluated as exploratory analyses.

The aim of this study was to determine whether baseline depressive symptoms are associated with incident frailty in older adults with cardiometabolic diseases and whether comorbidities modify this association. We hypothesized that the coexistence of depressive symptoms and diabetes would synergistically increase the risk of incident frailty. Further studies are needed to clarify the complex relationships between depressive symptoms, comorbidities, and the development of frailty and to determine whether targeted interventions can reduce the risk of frailty in this population.

## Methods

### Participants

Patients aged 65 years or older with cardiometabolic diseases who were referred to the frailty clinic [20] from the Department of Diabetes, Metabolism, and Endocrinology or Cardiology between July 29, 2015, and June 30, 2023 (data cutoff date) were eligible for inclusion. Enrollment is ongoing. Patients who completed at least one follow-up assessment were enrolled in this longitudinal study. Clinical psychologists conducted comprehensive geriatric assessments at baseline, including assessments of frailty, depressive symptoms, physical activity, social networks, and cognitive function. Patients diagnosed with dementia and those with frailty according to the modified Cardiovascular Health Study (mCHS) criteria [10,20] or the Kihon Checklist (KCL) [21] at baseline were excluded. Participants with incomplete baseline frailty assessments were also excluded from the analysis. The participants were followed up annually to determine the incidence of frailty until June 30, 2024.

### Frailty assessment

Using the mCHS [10,20] and KCL [21] criteria, we assessed two types of frailty; physical frailty and frailty based on a comprehensive geriatric assessment, respectively.

The mCHS criteria are criteria of the modified Japanese version of the Cardiovascular Health Study. The questions involved fatigue, lower physical activity—a point was added if either of the following two questions were applicable: “Do you go out less than once a week?” and “Have you gone out less often than last year?”—low walking speed (<1.0 m/second), decreased muscle strength (a grip strength of <28.0 kg for men and <18.0 kg for women), and weight loss (a loss of more than 2–3 kg in 6 months). In addition to the questionnaire, the 4-m walking speed and grip strength tests included in the Cardiovascular Health Study criteria were measured twice and once on each side, respectively, with the maximum value being adopted. Frailty was defined as an mCHS score of 3 or more.

The KCL is a frailty scale based on a comprehensive geriatric assessment for older adults. Specifically, it consists of 25 questions related to instrumental activities of daily living, locomotion, malnutrition, oral health, confinement, memory loss, and mental health. In this study, frailty was defined as a KCL score of 8 points or more [21].

### Depressive symptoms

The Geriatric Depression Scale (GDS)-15 was used to screen for depressive symptoms [22]. The cutoff value for depression is much debated, with some suggesting a value of 5 points or higher [23] and others suggesting one of 6 points or higher [22]. In this study, we adopted a cutoff value of 5 points or higher because this is the value most commonly used in clinical practice in Japan; the Japan Geriatrics Society share this value as a guideline for treating older patients [24].

### Social networks

Social networks were assessed using the Lubben Social Network Scale-6, which consists of questions about the number of family, relatives, and friends [25,26]. The total number of people is rated as 0 points for 0 people, 1 for 1 person, 2 for 2 people, 3 for 3–4 people, 4 for 5–8 people, and 5 for 9 people or more. The total score is calculated by summing the scores of the six items.

### Physical activity

Physical activity was assessed using the International Physical Activity Questionnaire-Short Form, which is used to evaluate the frequency of low, moderate, and vigorous physical activity [27]. The intensity of activity is expressed in metabolic equivalents (METs): 3.3 METs are allocated for walking, 4.0 METs for moderate physical activity, and 8.0 METs for vigorous physical activity. Each physical activity is expressed in METs × minutes, and the total weekly value is calculated. Total physical activity, the sum of three physical activities for 1 week, was used in this study.

### Cognitive function

The Japanese version of the Montreal Cognitive Assessment (MoCA-J), which is useful for screening mild cognitive impairment [28], was used to assess cognitive function. Cognitive impairment was defined as the MoCA-J of ≤23.

### Demographic and medical information

Age, sex, body mass index (BMI), number of years of education, number of medications taken; and diagnosis of hypertension, dyslipidemia, diabetes mellitus, heart failure, and atrial fibrillation from medical records were assessed at baseline. Polypharmacy was defined as taking at least five types of medication. The levels of glycated hemoglobin (HbA1c), lipids, and other biochemical data were measured during outpatient visits. Participants with diabetes were stratified by their baseline glycated hemoglobin (HbA1c) levels to examine the effect of hyperglycemia on frailty risk.

Hyperglycemia was defined as the HbA1c level of ≥ 6.5%, which corresponds to the diagnostic criterion for diabetes according to the American Diabetes Association [29]. The sensitivity analysis using an alternative threshold (HbA1c: 7.0%) was also performed to confirm the robustness of the findings. The HbA1c level of <7.0% was recommended as the glycemic goal for older adults with no functional limitations and minimal risk of severe hypoglycemia [30].

### Statistical analysis

First, data are described as means ± standard deviations or frequencies with percentages. We compared the characteristics of patients who developed frailty and those who did not. Differences in the numerical values between the two groups were assessed using t-tests. Differences in categorical values between the two groups were evaluated using the chi-square test.

Second, multiple Cox regression analysis was performed on all patients to determine whether depressive symptoms were independently associated with incident frailty. The independent variables were checked for multicollinearity by calculating the variance inflation factor. For sensitivity analysis, we performed the same analysis, but excluded patients with a follow-up period of less than 1 year and confirmed that the same results were obtained. As an additional sensitivity analysis, antidepressant use was additionally included as a covariate in the Cox regression models.

Model 1 was adjusted for age and sex; Model 2 was further adjusted for body mass index, number of years of education, number of medications, diabetes, hypertension, and dyslipidemia; and Model 3 was further adjusted for Lubben Social Network Scale-6 scores, MoCA-J scores, and total physical activity. A Kaplan–Meier curve was used to draw a frailty-free survival curve for the incidence of frailty. We compared the incidence of frailty between patients with and without depressive symptoms at baseline using a log-rank test.

Third, to examine the effect of the combination of depressive symptoms with comorbidities or clinical conditions on incident frailty, stratified analyses were performed using multiple Cox regression analyses. We performed a Cox regression analysis stratified by diabetes, hypertension, dyslipidemia, cognitive impairment, polypharmacy (≥5 types of medications), physical inactivity, and poor social support. To determine whether an interaction was present, a Cox regression analysis was performed using sex, age, stratified factors, depressive symptoms, and interaction terms for the risk of frailty according to the mCHS or KCL. The P-values for the interaction terms were then calculated.

Fourth, to examine the effects of a combination of depression and diabetic complications or medications on incident frailty in patients with diabetes, stratified analyses were performed using multiple Cox regression analyses on variables showing significant differences in the univariate analyses.

Finally, Kaplan–Meier curves and log-rank tests were used to compare the differences in incident frailty among the four groups: diabetes and depressive symptoms, depressive symptoms alone, diabetes alone, and neither diabetes nor depressive symptoms. IBM SPSS Statistics for Windows version 25.0 (IBM Corp., Armonk, NY, USA) was used for all analyses. Statistical significance was set at a P-value of less than 0.05. Missing data were assessed for all variables included in the analyses. Complete case analysis was used for each statistical model. Because different variables were included in each model, the number of participants varied across analyses depending on data availability. Complete-case analysis was performed for all regression models. Participants with missing data for any variable included in a given model were excluded from that analysis.

### Ethical considerations

All patients and their companions were given sufficient explanations and written consent was obtained. This study was approved by the Ethics Committee of the Tokyo Metropolitan Geriatric Hospital (approval number R15-20).

## Results

Of the 891 patients aged 65 years or older with cardiometabolic diseases who first visited the frailty clinic, 606 patients who visited the clinic at least twice were registered as participants. At baseline, we excluded 15 patients with dementia, 134 patients with frailty according to the mCHS criteria, and 190 patients according to the KCL criteria, respectively. Of these, 4 participants diagnosed according to the KCL criteria and 167 participants diagnosed according to the mCHS criteria could not be measured and were excluded as missing values. The patients included in this study were followed-up for a median period of 2.9 years. Finally, data from 431 participants was included in the analysis (Fig 1).

**Fig 1:**
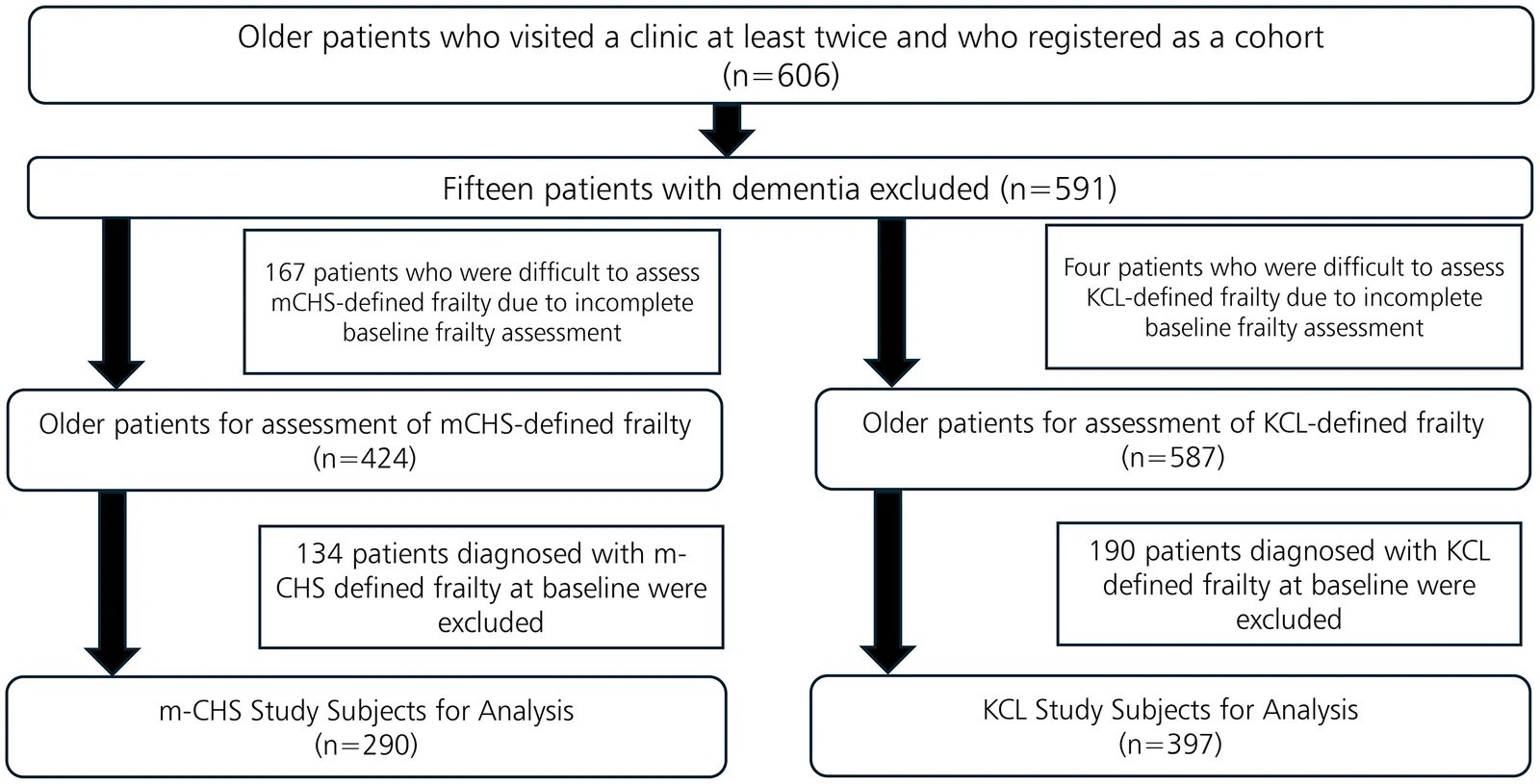
Flowchart of participant selection. mCHS, modified Cardiovascular Health Study; KCL, Kihon Checklist.

The mCHS and KCL analytical samples were not mutually exclusive. The overall study population consisted of 431 unique participants. Sample sizes in the Cox regression analyses were further reduced because complete-case analysis was applied.

Table 1 presents the baseline characteristics of the overall study population (n = 431). Separate analyses were subsequently conducted using the mCHS and KCL criteria, as shown in Fig 1. The mean age of the participants was 77.4 ± 6.3 years and 37.6% were men. The prevalences of diabetes, dyslipidemia, hypertension, heart failure, and atrial fibrillation were 51.0%, 65.7%, 74.2%, 5.8%, and 8.8%, respectively. The mean mCHS and KCL values were 1.1 ± 0.9 and 4.5 ± 2.5, respectively. The GDS-15 and MoCA-J scores were 3.2 ± 2.4 and 22.1 ± 4.1, respectively (Table 1).

**Table 1:** Patient characteristics in those who developed mCHS- or KCL-defined frailty and those who did not.

	Overall (n = 431)	Incidence of mCHS-defined frailty (+) (n = 91)	Incidence of mCHS-defined frailty (-) (n = 199)	P-value	Incidence of KCL-defined frailty (+) (n = 92)	Incidence of KCL-defined frailty (-) (n = 305)	P-value
Age (years)	77.4 ± 6.3	**79.9 ± 5.9**	**76.6 ± 6.4**	**<0.001**	**79.2 ± 5.2**	**76.8 ± 6.5**	**0.001**
Sex (Men)	37.6%	35.2%	41.7%	0.290	**28.09%**	**40.14%**	**0.040**
BMI (kg/m^2^)	23.4 ± 3.6	**24.2 ± 4.7**	**22.9 ± 4.3**	**0.016**	22.5 ± 5.9	23.4 ± 3.6	0.070
Education (years)	12.6 ± 2.7	**11.3 ± 2.4**	**12.8 ± 2.7**	**<0.001**	**11.4 ± 2.6**	**12.9 ± 2.7**	**<0.001**
Number of drugs	5.3 ± 3.2	**6.1 ± 2.7**	**5.2 ± 3.2**	**0.013**	**6.6 ± 3.2**	**4.8 ± 3.2**	**<0.001**
**Comorbidity**							
Diabetes	51.0%	57.1%	51.8%	0.394	53.26%	48.30%	0.406
Dyslipidemia	65.7%	64.8%	70.4%	0.348	65.9%	64.3%	0.774
Hypertension	74.2%	73.6%	78.4%	0.372	82.2%	73.5%	0.091
Heart failure	5.8%	5.5%	5.1%	0.873	8.0%	5.5%	0.402
Atrial fibrillation	8.8%	8.9%	9.6%	0.849	10.1%	9.0%	0.744
HbA1c (≥6.5%)	46.2%	45.1%	46.2%	0.852	50.0%	44.3%	0.344
HbA1c (%)	6.4 ± 1.3	6.5 ± 1.4	6.4 ± 1.3	0.597	6.5 ± 1.6	6.3 ± 1.3	0.228
LDL-C (mg/dl)	108 ± 31	105 ± 27	110 ± 31	0.182	107 ± 27	110 ± 30	0.534
TG (Mg/dL)	133 ± 71	137 ± 63	136 ± 72	0.897	129 ± 54	135 ± 74	0.510
HDL-C (mg/dl)	59 ± 17	**55 ± 14**	**59 ± 18**	**0.042**	58 ± 16	59 ± 17	0.761
Systolic blood pressure (mmHg)	130 ± 21	127 ± 17	132 ± 19	0.053	129 ± 24	130 ± 23	0.639
Diastolic blood pressure (mmHg)	74 ± 14	**71 ± 14**	**75 ± 11**	**0.006**	71 ± 14	74 ± 15	0.120
Antidepressant	3.7%	7.1%	2.6%	0.091	4.7%	3.5%	0.630
**Outcomes (Frailty)**							
mCHS (points)	1.1 ± 0.9	**1.4 ± 0.8**	**0.8 ± 0.8**	**<0.001**	**1.6 ± 1.1**	**0.9 ± 0.8**	**<0.001**
KCL (points)	4.5 ± 2.5	**6.8 ± 3.1**	**4.5 ± 3.0**	**<0.001**	**5.1 ± 1.8**	**3.7 ± 1.9**	**<0.001**
**Measurement items**							
GDS-15 (points)	3.2 ± 2.4	**4.3 ± 2.6**	**3.4 ± 2.6**	**0.005**	**4.0 ± 2.4**	**2.8 ± 2.2**	**<0.001**
MoCA-J (points)	22.1 ± 4.1	**20.3 ± 3.9**	**22.4 ± 4.2**	**<0.001**	**20.5 ± 4.4**	**22.5 ± 4.1**	**<0.001**
Physical Activity (MET minutes/week)	2,758 (1,200−4,927)	**1,840** **(930−3,570)**	**3,360** **(1,440−5,040)**	**0.003**	2,880 (960−4,480)	2,834 (1,420−4,994)	0.421
LSNS-6 (points)	13.8 ± 5.9	12.0 ± 5.7	13.3 ± 5.8	0.089	13.5 ± 5.8	14.2 ± 5.8	0.325

Data are presented as means±standard deviations or medians (interquartile ranges). A t-test was used for continuous variables, a χ-square test for qualitative variables and a Mann-Whitney U-test for non-normally distributed physical activity measures. The t-test was two-tailed. The χ-square test was two-tailed with the asymptotic significance probability (two-tailed) of Pearson’s chi-square.

Means and standard deviations are presented for continuous variables, percentages for qualitative variables, and medians and interquartile ranges for physical activity.

All variables represent baseline characteristics. + indicates onset, – indicates no onset.

mCHS, modified Cardiovascular Health Study; KCL, Kihon Checklist

The characteristics of the participants were compared between those who developed frailty during follow-up and those who did not (Table 1). Those who developed mCHS-or KCL-defined frailty had higher GDS-15 score, a large number of medications, older age, fewer years of education, and lower MoCA-J scores than those who did not.

### Kaplan–Meier curve analysis

Fig 2 shows the Kaplan–Meier curve for frailty-free survival in patients with and without depressive symptoms at baseline.

**Fig 2:**
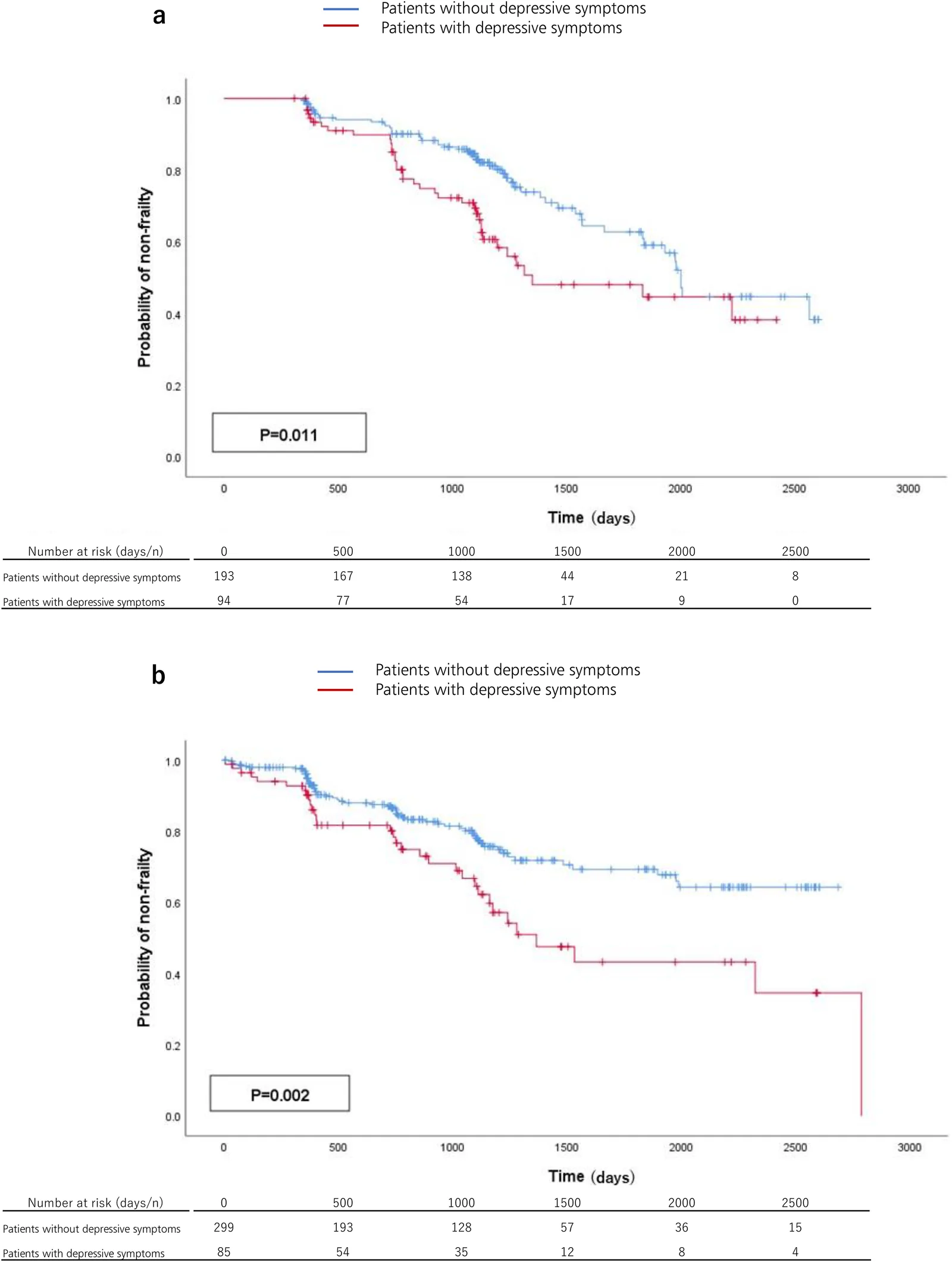
Frailty-free survival curves among patients with and without depressive symptoms at baseline. **a.** Frailty defined using the modified Cardiovascular Health Study (mCHS) criteria. During the follow-up period, 37 (39.3%) patients with depressive symptoms and 53 (27.5%) without had mCHS-defined frailty. The difference in incident frailty between the two groups was significant (P = 0.011; log-rank test). **b.** Frailty defined using the Kihon Checklist (KCL) criteria. During the follow-up period, 32 (37.6%) patients with depressive symptoms and 59 (19.7%) without had KCL-defined frailty. The difference in incident frailty between the two groups was significant (P = 0.002; log-rank test).

According to the mCHS and KCL criteria, those with depressive symptoms were more likely to have incident frailty than those without (P = 0.011 and 0.002, respectively; log-rank test).

### Multiple Cox regression analyses

Multiple Cox regression analyses were performed on all patients to determine whether depressive symptoms were independently associated with incident frailty. An independent association between depressive symptoms and the incidence of mCHS- or KCL-defined frailty was observed in all three models after adjusting for covariates (Table 2 and Table 3). The hazard ratios (HRs) of depressive symptoms for incident mCHS-defined frailty were 1.93 (95% confidence interval [CI]: 1.26–2.95) in Model 1, 1.89 (95% CI: 1.18–3.01) in Model 2, and 1.92 (95% CI: 1.13–3.25) in Model 3 (Table 2). In Model 3, age, higher BMI, polypharmacy, lower physical activity, and a cognitive impairment were associated with incident frailty.

**Table 2:** Multiple Cox regression analysis for the association between depressive symptoms and incident mCHS-defined frailty.

	Model 1		Model 2		Model 3	
**Variables**	HR (95%CI)	P-value	HR (95%CI)	P-value	HR (95%CI)	P-value
**Independent Variable**						
GDS-15 (≥5 points)	**1.93(1.26-2.95)**	**0.002**	**1.89(1.18-3.01)**	**0.008**	**1.92(1.13-3.25)**	**0.016**
**Adjustment Variables**						
Age	**1.12(1.08-1.16)**	**<0.001**	**1.12(1.07-1.18)**	**<0.001**	**1.11(1.06-1.16)**	**<0.001**
Sex (Men)	0.95(0.61-1.46)	0.800	0.80(0.49-1.30)	0.373	0.67(0.39-1.11)	0.132
Education			**0.90(0.81-0.99)**	**0.028**	0.91(0.82-1.00)	0.054
Polypharmacy (5 ≥ types)			**1.87(1.09-3.21)**	**0.023**	**2.02(1.17-3.47)**	**0.011**
BMI			**1.09(1.02-1.17)**	**0.009**	**1.08(1.01-1.15)**	**0.026**
Dyslipidemia			0.75(0.46-1.23)	0.260	0.82(0.50-1.36)	0.440
Diabetes			1.11(0.68-1.82)	0.676	1.19(0.72-1.98)	0.493
Hypertension			0.88(0.53-1.48)	0.640	0.81(0.48-1.38)	0.446
Social network (<Median)					0.93(0.55-1.59)	0.798
Cognitive impairment					**1.77(1.04-3.00)**	**0.036**
Physical Activity (>Median)					**0.62(0.38-0.99)**	**0.046**

The number of cases used in the analysis was 286, 262, and 256 in Models 1, 2, and 3, respectively.

The median values for social networks and physical activity were 13 and 2400, respectively.

Model 1: adjusted for age and sex; Model 2: further adjusted for BMI, years of education, number of medications, diabetes, hypertension, and dyslipidemia; Model 3: further adjusted for LSNS-6 scores, MoCA-J scores, and total physical activity.

HR, hazard ratio; CI, confidence interval; BMI, body mass index.

**Table 3:** Multiple Cox regression analysis for the association between depressive symptoms and incident KCL-defined frailty.

Variables	Model 1		Model 2		Model 3	
	HR (95%CI)	P-value	HR (95%CI)	P-value	HR (95%CI)	P-value
**Independent Variable**						
GDS-15 (≥5 points)	**2.29(1.48-3.57)**	**<0.001**	**1.97(1.21-3.21)**	**0.006**	**2.05(1.23-3.42)**	**0.006**
**Adjustment Variables**						
Age	**1.09(1.05-1.13)**	**<0.001**	**1.07(1.02-1.11)**	**0.004**	**1.06(1.01−1.11)**	**0.011**
Sex (Men)	0.77(0.48-1.23)	0.293	0.85(0.52-1.39)	0.507	0.77(0.45-1.32)	0.346
Education			0.92(0.83-1.01)	0.092	0.94(0.85-1.03)	0.181
Polypharmacy (5 ≥ types)			**1.82(1.10-3.02)**	**0.021**	**1.86(1.10-3.13)**	**0.021**
BMI			0.95(0.89-1.00)	0.066	0.95(0.90-1.00)	0.071
Dyslipidemia			1.04(0.64-1.67)	0.889	1.13(0.69-1.85)	0.641
Diabetes			0.92(0.57-1.48)	0.732	0.93(0.57-1.51)	0.768
Hypertension			1.53(0.85-2.75)	0.157	1.43(0.77-2.63)	0.258
Social network (<Median)					1.46(0.92-2.32)	0.112
Cognitive impairment					**2.01(1.20-3.37)**	**0.008**
Physical Activity (>Median)					0.87(0.55-1.39)	0.561

Model 1: adjusted for sex and age; Model 2: further adjusted for baseline BMI, years of education, number of medications, diabetes, hypertension, and dyslipidemia; Model 3; further adjusted for LSNS-6 scores, MoCA-J scores, and total physical activity.

The number of cases used in the analysis were 382, 322, and 297 in Models 1, 2, and 3, respectively. The median values for social networks and physical activity were 14 and 2880, respectively. KCL, Kihon Checklist; HR, hazard ratio; CI, confidence interval; BMI, body mass index

The HRs of depressive symptoms for incident KCL-defined frailty were 2.29 (95% CI: 1.48–3.57) in Model 1, 1.97 (95% CI: 1.21–3.21) in Model 2, and 2.05 (95% CI: 1.23–3.42) in Model 3 (Table 3). In Model 3, age, polypharmacy and a cognitive impairment were independent predictors of incident KCL-defined frailty.

In sensitivity analyses excluding participants with a follow-up period of less than 1 year, depressive symptoms remained significantly associated with incident mCHS-defined frailty (HR, 1.87; 95% CI, 1.20–2.89; P = 0.005) and KCL-defined frailty (HR, 2.34; 95% CI, 1.40–3.90; P = 0.001). Similarly, after additionally adjusting for antidepressant use, depressive symptoms remained significantly associated with incident mCHS-defined frailty (HR, 1.90; 95% CI, 1.23–2.95; P = 0.004) and KCL-defined frailty (HR, 2.06; 95% CI, 1.30–3.27; P = 0.002). Detailed results are presented in Supplementary Table 1.

### Stratified analyses

Stratified analyses according to diabetes, hypertension, dyslipidemia, cognitive impairment, polypharmacy, physical activity, and poor social network were performed using the Cox regression model in Model 1 (Table 4). Depressive symptoms were significantly associated with incident mCHS-defined frailty in participants with diabetes, HbA1c ≥ 6.5%, cognitive impairment, and low physical activity. No significant associations were observed in the corresponding subgroups without diabetes or cognitive impairment, or with higher physical activity levels. Regarding frailty defined using the KCL criteria, depressive symptoms were significantly associated with incident frailty in participants with diabetes and those with HbA1c ≥ 6.5% or ≥7.0% (Table 5). No significant associations were observed in the subgroups stratified by hypertension, dyslipidemia, cognitive impairment, polypharmacy, or physical activity.

**Table 4:** Stratified analysis for the association between the combination of depression with comorbidity, cognitive impairment, polypharmacy, physical activity, and social network and incidence of mCHS-defined frailty.

Conditions in combination with depressive symptoms	n	HR (95%CI)	P-value	Interaction P
Diabetes (+)	150	**3.18(1.82-5.54)**	**<0.001**	**0.016**
Diabetes (-)	134	1.06(0.53-2.11)	0.876	
HbA1c (≥7.5%)	42	3.20(0.94-10.89)	0.063	0.465
HbA1c (<7.5%)	243	**1.80(1.13-2.85)**	**0.013**	
HbA1c (≥7.0%)	85	1.85(0.84-4.04)	0.125	0.927
HbA1c (<7.0%)	200	**1.97(1.18-3.28)**	**0.009**	
HbA1c (≥6.5%)	166	**3.08(1.65-5.74)**	**<0.001**	0.059
HbA1c (<6.5%)	155	1.36(0.5-2.46)	0.306	
Hypertension (+)	219	**1.99(1.21-3.26)**	**0.006**	0.770
Hypertension (-)	64	**2.53(1.00-6.36)**	**0.049**	
Dyslipidemia (+)	195	1.64(0.94-2.87)	0.084	0.372
Dyslipidemia (-)	90	**2.50(1.22-5.13)**	**0.012**	
Cognitive impairment (+)	168	**2.33(1.41-3.86)**	**0.001**	0.281
Cognitive impairment (-)	117	1.50(0.66-3.43)	0.336	
Polypharmacy (+)	170	1.63(0.96-2.77)	0.070	0.285
Polypharmacy (-)	115	**2.38(1.11-5.11)**	**0.026**	
Physical activity (≥median)	145	1.56(0.77-3.16)	0.220	0.888
Physical activity (<median)	131	**2.07(1.18-3.62)**	**0.011**	
Social network (≥median)	128	**2.58(1.03-6.46)**	**0.043**	0.839
Social network (<median)	156	1.66(0.98-2.82)	0.059	

These analyses were performed using the Cox regression analysis adjusted for sex and age.

The median values for social networks and physical activity were 13 and 2400, respectively.

For the two stratified groups, if both were significant or there was a combination of significant trends, there was no apparent difference between the two groups.

+ indicates onset, – indicates no onset. mCHS, modified Cardiovascular Health Study; HR, hazard ratio; CI, confidence interval

**Table 5:** Stratified analysis for the association between the combination of depression with comorbidity, cognitive impairment, polypharmacy, physical activity, and social network and incidence of KCL-defined frailty.

Conditions in combination with depressive symptoms	n	HR (95%CI)	P-value	Interaction P
Diabetes (+)	188	**3.72(2.03-6.80)**	**<0.001**	**0.023**
Diabetes (-)	192	1.36(0.68-2.72)	0.384	
HbA1c (≥7.5%)	50	2.40(0.73-7.96)	0.152	0.803
HbA1c (<7.5%)	329	**2.22(1.35-3.65)**	**0.002**	
HbA1c (≥7.0%)	112	**4.32(2.01-9.28)**	**<0.001**	0.069
HbA1c (<7.0%)	267	1.75(0.99-3.11)	0.056	
HbA1c (≥6.5%)	171	**4.01(2.13-7.55)**	**<0.001**	**0.016**
HbA1c (<6.5%)	208	1.36(0.71-2.61)	0.35	
Hypertension (+)	286	**2.23(1.36-3.64)**	**0.001**	0.698
Hypertension (-)	94	**3.31(1.05-10.48)**	**0.041**	
Dyslipidemia (+)	245	**2.11 (1.17-3.81)**	**0.014**	0.304
Dyslipidemia (-)	136	**3.53(1.66-7.47)**	**0.001**	
Cognitive impairment (+)	223	**1.79(1.05-3.06)**	**0.033**	0.195
Cognitive impairment (-)	153	**3.76(1.67-8.47)**	**0.001**	
Polypharmacy (+)	199	**1.93(1.12-3.33)**	**0.018**	0.387
Polypharmacy (-)	182	**2.57(1.18-5.57)**	**0.017**	
Physical activity (≥median)	170	**2.07(1.09-3.95)**	**0.027**	0.929
Physical activity (<median)	152	**2.56(1.34-4.88)**	**0.004**	
Social network (≥median)	175	**3.50(1.78-6.85)**	**<0.001**	0.168
Social network (<median)	207	1.71(0.95-3.11)	0.076	

These analyses were performed using the Cox regression analysis adjusted for sex and age.

The median values for social networks and physical activity were 14 and 2880, respectively.

For the two stratified groups, if both were significant or there was a combination of significant trends, there was no apparent difference between the two groups.

+ indicates onset, – indicates no onset.

KCL, Kihon Checklist; HR, hazard ratio; CI, confidence interval

The interaction between depressive symptoms and diabetes or hyperglycemia on incident frailty was examined using Cox regression models adjusted for age and sex (Tables 4 and 5). A significant interaction between depressive symptoms and diabetes was observed for both mCHS-defined frailty (P for interaction = 0.016) and KCL-defined frailty (P for interaction = 0.023). In addition, a significant interaction between depressive symptoms and HbA1c ≥ 6.5% was observed for KCL-defined frailty (P for interaction = 0.016). No significant interactions were observed for the other stratification variables.

Kaplan–Meier analysis showed that patients with a combination of depressive symptoms and diabetes were most likely to develop both types of frailty (Fig 3). The difference in the incidence of mCHS- and KCL-defined frailty among the four groups was significant (P = 0.002 and P = 0.004, respectively).

**Fig 3:**
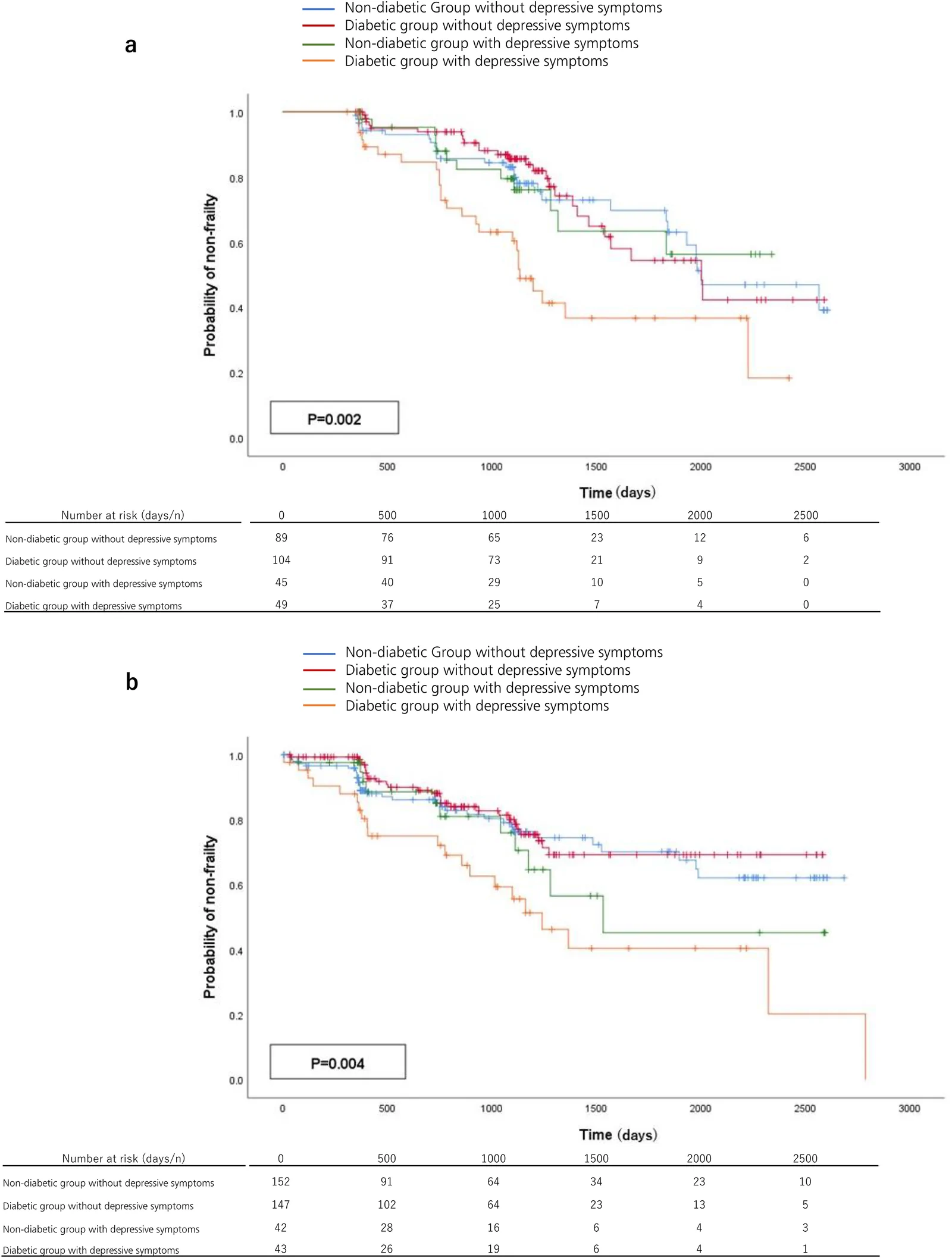
Frailty-free survival curves among the four groups according to the presence of depressive symptoms and diabetes. **a.** Frailty defined according to the modified Cardiovascular Health Study (mCHS) criteria. During the follow-up period, 27 (30.3%) patients in the non-diabetic group and 26 (25.0%) in the diabetic group with no depressive symptoms as well as 12 (26.6%) in the non-diabetic group and 25 (51.0%) in the diabetic group with depressive symptoms developed mCHS-defined frailty. The differences among the four groups were significant (P = 0.002; log-rank test). **b.** Frailty defined according to the Kihon Checklist (KCL) criteria. During the follow-up period, 32 (21.1%) patients in the non-diabetic group and 27 (18.3%) in the diabetic group with no depressive symptoms as well as 11 (26.1%) in the non-diabetic group and 21 (48.8%) in the diabetic group with depressive symptoms developed KCL-defined frailty. The differences among the four groups were significant (P = 0.004; log-rank test).

### Combination effect of diabetic complications or medications and depression on frailty

The frequency of complications among patients with diabetes was as follows: angina pectoris, 18.5%; myocardial infarction, 7.3%; stroke, 19.5%; peripheral arterial disease, 5.0%; and nephropathy, 55.5%. The prevalences of these complications at baseline were similar among those who developed mCHS-defined and KCL-defined frailty; however, those who developed KCL-defined frailty had a higher prevalence of stroke. Medications among patients with diabetes were as follows: DPP (dipeptidyl peptidase)-4 inhibitors, 68.2%; SGLT2 (sodium-glucose cotransporter 2) inhibitors, 11.4%; metformin, 52.7%; sulfonylureas (SU), 25.0%; glinides, 8.2%; GLP-1 (glucagon-like peptide-1) receptor agonists, 3.2%; and insulin therapy, 6.8%. The proportion of users of SU drugs and insulin was higher among those who developed KCL-defined frailty than among those who did not (Supplementary Table 2). As an exploratory analysis, multivariable Cox regression analyses of diabetic complications and diabetes medications were performed only for KCL-defined frailty because significant differences in these variables were observed only in the baseline comparison for KCL-defined frailty. Neither SU drug use (HR, 1.35; 95% CI, 0.68–2.67; P = 0.393) nor insulin therapy (HR, 1.75; 95% CI, 0.83–3.70; P = 0.142) was independently associated with incident KCL-defined frailty (Supplementary Table 3).

## Discussion

In this longitudinal study, depressive symptoms were independently associated with the incidence of two types of frailty in older outpatients with cardiometabolic diseases after adjusting for comorbidities, cognitive impairment, polypharmacy, and social networks. Importantly, we found a significant interaction between depressive symptoms and diabetes, suggesting that depressive symptoms had a stronger association with incident frailty among patients with diabetes than among those without diabetes. These findings support our hypothesis that the coexistence of depressive symptoms and diabetes synergistically increases the risk of incident frailty. Our results are consistent with those of another longitudinal study involving community-dwelling older adults [11] and extend our previous cross-sectional findings in older outpatients with cardiometabolic diseases [13]. We examined the effects of the combination of depressive symptoms with cardiometabolic disease on incident frailty. The stratified analysis showed a significant interaction between depressive symptoms and diabetes on the incidence of frailty defined according to both the mCHS and KCL criteria. In fact, the risk of frailty in patients with diabetes and depressive symptoms was 3.18 to 3.72 times higher than that in patients without depressive symptoms, whereas depressive symptoms had a similar effect on the onset of frailty regardless of whether hypertension or dyslipidemia was present.

The presence of diabetes mellitus was the only factor that interacted with depression in the stratified analysis. Furthermore, we found a synergistic effect between an HbA1c level of ≥6.5% and depressive symptoms on incident frailty. Our findings differ from those of Zaslavsky et al. [31], who reported a U-shaped association between average glucose levels and incident frailty among older adults with diabetes. In their study, average glucose levels corresponding approximately to HbA1c levels of 6.9% and 8.2% were associated with an increased risk of frailty. Low HbA1c levels have been shown to be a risk factor for frailty in older people with diabetes [32]. In one longitudinal study, an HbA1c level of 8.0% or more was associated with the incidence of frailty in older women [33]. This discrepancy may be due to age, glucose control, diabetes treatment, and the setting of the study population. In the present study, most older outpatients with diabetes were treated according to the glycemic targets recommended by the Japan Diabetes Society and the Japan Geriatrics Society and generally had well-controlled glucose levels without apparent hypoglycemia. Therefore, the subgroup with HbA1c ≥ 6.5% should not be interpreted as representing poor glycemic control but rather as participants meeting the diagnostic threshold for diabetes. The burden of diabetes treatment, even among patients with relatively well-controlled diabetes, may also have contributed to the observed association with incident frailty. Our results suggest that the predisposition of older adults with diabetes and depression to frailty should be given particular attention. Early screening, prevention, and intervention for depressive symptoms are necessary to prevent frailty in older patients with diabetes. As an exploratory analysis, we further examined the associations of diabetic complications and diabetes medications with incident frailty among participants with diabetes. Because significant differences in the prevalence of stroke and the use of SU drugs and insulin were observed only for KCL-defined frailty in the baseline comparison (Supplementary Table 2), multivariable Cox regression analyses were performed only for KCL-defined frailty. In these analyses, neither stroke (HR,1.63; 95% CI, 0.76–3.53; P = 0.211), SU drug use (HR, 1.35; 95% CI, 0.68–2.67; P = 0.393) nor insulin therapy (HR, 1.75; 95% CI, 0.83–3.70; P = 0.142) was independently associated with incident KCL-defined frailty (Supplementary Table 3).

Diabetes and depression may share common etiologies or pathways that lead to frailty: chronic inflammation, insulin resistance, and increased cortisol secretion resulting from activation of the hypothalamic–pituitary–adrenal (HPA) axis [18, 34]. Both hyperglycemia and depression can cause chronic stress, leading to overactivation of the hypothalamic–pituitary–adrenal axis, leading to increased secretion of inflammatory cytokines and cortisol [34], which may contribute to physical frailty [35] by promoting muscle degradation [36]. Furthermore, the insulin resistance in depression as well as diabetes may contribute to the onset of frailty through mechanisms such as muscle breakdown [37, 38]. Other psychosocial factors such as distress, negative emotions, and stigma in patients with diabetes with depression may impair their ability to engage in activities of self-care, such as exercise, diet, and drug adherence [14], leading to the onset of frailty through hyperglycemia and diabetic complications.

In the present study, cognitive impairment and polypharmacy were independently associated with the incidence of frailty; this finding is consistent with those of previous studies [39, 40]. Additionally, the combination of depressive symptoms with cognitive impairment was associated with frailty defined according to the mCHS criteria, which is consistent with the findings of a Chinese oldest old population [41]. Both conditions may demonstrate similar neurological changes—such as white matter lesions—that accelerate the incidence of frailty. Patients with major depressive disorder and mild cognitive impairment share volumetric reductions in the insula and superior temporal gyrus, which result in decreased participation in mental or social activities, thus leading to frailty [42]. Depressive symptoms, mild cognitive impairment, and orthostatic hypotension, however, may be precursors to Lewy body dementia [43]. Older adults with cardiometabolic diseases are more likely to develop mild cognitive impairment [44,45], orthostatic hypotension [46], and depression, all of which—when present together—lead to frailty. These findings suggest that more attention should be paid to the incidence of frailty in older adults with mild cognitive impairment and depressed mood.

In the present study, we found that the combination of depressive symptoms with physical inactivity was associated with mCHS-defined frailty. This is consistent with the findings of previous studies that reported that physical inactivity and depressive symptoms are more likely to lead to frailty when combined [47]. However, since our study did not find a significant interaction between depressive symptoms and reduced physical activity, further research is needed.

Our results suggest that depressive symptoms, cognitive impairment, and reduced physical activity affect the onset of frailty and that the combination of depression with diabetes may accelerate the incidence of frailty. This supports the notion that comprehensive geriatric assessment and individualized treatment are imperative in older adults with cardiometabolic diseases such as diabetes.

The strengths of the present study include its use of a longitudinal follow-up design and its relatively large sample size of older outpatients with cardiometabolic diseases (e.g., diabetes, hypertension, and dyslipidemia) at high risk of frailty. The study examined the incidence of two types of frailty as an outcome measure.

This study had several limitations. First, it was conducted at a single center, and the generalizability of the findings should be confirmed in future multicenter studies. Second, because this study included relatively health-conscious older adults with cardiometabolic diseases who were at risk of frailty, selection bias may have influenced the results. Third, only participants who completed at least one follow-up assessment were included in the longitudinal analysis. Therefore, participants who were lost to follow-up after the baseline assessment, those with dementia at baseline, and those who could not complete the baseline frailty assessment were excluded, which may have introduced healthy-participant bias. Fourth, complete-case analysis was used for all regression models. Consequently, the sample size varied across models because participants with missing values for variables included in each model were excluded from the corresponding analysis. Therefore, bias cannot be excluded if the missing data were not completely random. In addition, multiple imputation was not performed, and the findings should be interpreted with caution. Fifth, frailty was assessed annually; therefore, the exact time of frailty onset was not observed, and the Cox regression analyses treated the event time as the follow-up assessment at which frailty was first identified. Finally, the proportional hazards assumption of the Cox regression models was not formally evaluated. Finally, social determinants, such as economic status and caregiving environment, were not evaluated.

## Conclusion

In this longitudinal study, depressive symptoms were independently associated with the incidence of two types of frailty in older outpatients with cardiometabolic diseases. A significant interaction between depressive symptoms and diabetes was observed, whereas no significant interactions were found for cognitive impairment or physical inactivity. In addition, depressive symptoms in combination with hyperglycemia were associated with an increased risk of incident frailty. These findings suggest that older outpatients attending a frailty clinic for cardiometabolic diseases, particularly those with coexisting depressive symptoms and diabetes, may represent a high-risk group for incident frailty and may benefit from careful assessment of depressive symptoms and frailty risk. Further studies are needed to determine whether interventions targeting depressive symptoms and diabetes management can prevent frailty and to identify effective physical, cognitive, and psychosocial strategies for reducing frailty risk in this population.

### Patient Consent Statement

Informed consent was obtained from all individual participants included in the study. For patients recognized as cognitively impaired, proxy consent was obtained from a family member or another supportive adult on their behalf.

## Supporting information

S1 File Supplementary Tables 1–3. (DOCX)
