# Supplementary material for: Depressive symptoms are associated with the incidence of frailty in older adults with cardiometabolic diseases: Interaction between depression and diabetes
Source: PLoS One. 2026 Oct 26;21(10):e0360802. doi: 10.1371/journal.pone.0360802 (PMC13704878; doi:10.1371/journal.pone.0360802)
Supplement: Supplementary file 1 [file pone.0360802.s001.docx]

| Sensitivity analysis | Frailty definition | HR (95% CI) | P-value |
| --- | --- | --- | --- |
| Follow-up <1 year excluded | mCHS | 1.87 (1.20–2.89) | 0.005 |
| Follow-up <1 year excluded | KCL | 2.34 (1.40–3.90) | 0.001 |
| Additionally adjusted for antidepressant use | mCHS | 1.90 (1.23–2.95) | 0.004 |
| Additionally adjusted for antidepressant use | KCL | 2.06 (1.30–3.27) | 0.002 |

**Supplementary Table 1. Sensitivity analyses of the association between depressive symptoms and incident frailty**

Hazard ratios were adjusted for age and sex.

| **Supplementary Table 2. Frequency of Complications and Medications in Diabetic Patients** |  | Incidence of mCHS-defined frailty (+) (n=52) | Incidence of mCHS-defined frailty (-) (n=103) | P-value |  | Incidence of KCL-defined frailty (+) (n=49) | Incidence of KCL-defined frailty (-) (n=153) | P-value |
| --- | --- | --- | --- | --- | --- | --- | --- | --- |
| Complications (%) |  |  |  |  |  |  |  |  |
| Angina pectoris |  | 7.8 | 12.7 | 0.363 |  | 15.2 | 12.2 | 0.601 |
| myocardial infarction |  | 2.0 | 8.8 | 0.105 |  | 8.7 | 4.3 | 0.255 |
| stroke |  | 19.6 | 12.6 | 0.252 |  | **19.6** | **8.6** | **0.041** |
| peripheral arterial disease |  | 0.0 | 3.9 | 0.152 |  | 2.2 | 3.6 | 0.627 |
| nephropathy |  | 51.9 | 60.2 | 0.325 |  | 57.1 | 57.0 | 0.990 |
| Medication (%) |  |  |  |  |  |  |  |  |
| Biguanides |  | 58.8 | 49.0 | 0.252 |  | 62.2 | 48.2 | 0.102 |
| DPP-4 inhibitors |  | 68.6 | 71.8 | 0.679 |  | 63.8 | 66.9 | 0.700 |
| SGLT2 inhibitors |  | 13.7 | 6.8 | 0.159 |  | 15.9 | 9.9 | 0.268 |
| SU drugs |  | 31.4 | 27.2 | 0.588 |  | **37.0** | **21.1** | **0.031** |
| Glinides |  | 11.1 | 15.0 | 0.779 |  | 11.1 | 6.4 | 0.614 |
| GLP-1 receptor agonists |  | 5.8 | 3.0 | 0.406 |  | 2.2 | 3.6 | 0.647 |
| Insulin therapy |  | 7.7 | 7.0 | 0.876 |  | **19.6** | **4.4** | **0.001** |

KCL,Kihon Checklist; mCHS, modified Cardiovascular Health Study

+ indicates onset, - indicates no onset.

| **Supplementary Table 3. Multiple Cox Regression Analysis on the Association Between Depression Symptoms, Complications, and Medications and KCL-defined Frailty in Patients with Diabetes Mellitus** | | | | | | | | | |
| --- | --- | --- | --- | --- | --- | --- | --- | --- | --- |
|  | Model 1 |  |  | Model 2 |  |  | Model 3 |  | |
| **Variables** | HR (95%CI) | P-value |  | HR (95%CI) | P-value |  | HR (95%CI) | | P-value |
| **Independent Variable** |  |  |  |  |  |  |  | |  |
| GDS-15 (5 points or more) | **3.40(1.83-6.31)** | **<0.001** |  | **3.85(2.09-7.12)** | **<0.001** |  | **3.43(1.86-6.33)** | | **<0.001** |
| **Adjustment Variables** |  |  |  |  |  |  |  | |  |
| Age | **1.07(1.02-1.13)** | **0.006** |  | **1.07(1.01-1.13)** | **0.006** |  | **1.09(1.03-1.14)** | | **0.002** |
| Sex | 0.57(0.29-1.12) | 0.101 |  | 0.56(0.28-1.12) | 0.100 |  | 0.56(0.29-1.08) | | 0.084 |
| Stroke | 1.63(0.76-3.53) | 0.211 |  |  |  |  |  | |  |
| SU drug |  |  |  | 1.35(0.68-2.67) | 0.393 |  |  | |  |
| Insulin therapy |  |  |  |  |  |  | 1.75(0.83-3.70) | | 0.142 |

KCL, Kihon Checklist; HR, hazard ratio; CI, confidence interval
